# A hypothesis to derive the shape of the dose–response curve for teratogenic radiation effects

**DOI:** 10.1186/s12940-022-00837-z

**Published:** 2022-02-10

**Authors:** Alfred Körblein

**Affiliations:** Untere Söldnersgasse 8, 90403 Nürnberg, Germany

**Keywords:** Dose–response, Teratogenic effects, Monte Carlo simulation, Ionizing radiation

## Abstract

Reports of adverse pregnancy outcomes after in utero exposure to very low levels of ionizing radiation are inconsistent with a threshold dose of 100 mSv for teratogenic effects in humans. In the present study, it is hypothesized that the shape of the dose–response relationship for teratogenic effects is a cumulative lognormal distribution without threshold. This hypothesis relies on the assumption that both doses and radiosensitivities in human populations exposed to ionizing radiation are random variables, modeled by lognormal density functions. Here, radiosensitivity is defined as the dose limit up to which radiation damage can be repaired by the cellular repair systems, in short, the repair capacity. Monte Carlo simulation is used to generate N pairs of individual doses and repair capacities. Radiation damage occurs whenever the dose exceeds the related repair capacity. The rate of radiation damage is the number of damages, divided by the number N of pairs. Monte Carlo simulation is conducted for a sufficient number of ascending median doses. The shape of the dose–response relationship is determined by regression of damage rates on mean dose. Regression with a cumulative lognormal distribution function yields a perfect fit to the data. Acceptance of the hypothesis means that studies of adverse health effects following in-utero exposure to low doses of ionizing radiation should not be discarded primarily because they contradict the concept of a threshold dose for teratogenic effects.

**Supplementary Information:**

The online version contains supplementary material available at 10.1186/s12940-022-00837-z.

## Background

According to ICRP Publication 90 (2003) [1], teratogenic radiation effects, i.e. adverse health effects after in-utero exposure, are not expected to occur in human populations below a threshold dose of 100 mSv. The United Nations Scientific Committee on the Effects of Atomic Radiation (UNSCEAR) has published several reports on the health effects of the nuclear accidents at Chernobyl and Fukushima [2–4]. Annex J of the UNSCEAR 2000 [2] states that, after Chernobyl, “no changes in birth defects over time could be related to exposure to ionizing radiation.” And: “Congenital malformations, stillbirths, premature births, and perinatal mortality were studied, but no consistency or apparent relationship to ionizing radiation was noticed.” A search for “perinatal mortality” in the UNSCEAR 2008 report Annex D [3] on health effects due to radiation from the Chernobyl accident retrieved no results. The UNSCEAR 2013 report Annex A [4] on Fukushima health effects states that “prenatal exposure resulting from the accident at the Fukushima Daiichi nuclear power station (FDNPS) is not expected to increase the incidence of spontaneous abortion (miscarriages), perinatal mortality, congenital effects or cognitive impairment.”

An online guide by the U.S. Cencters for Disease Control and Prevention (CDC), titled “Radiation and Pregnancy: A Fact Sheet for Clinicians” and meant to provide physicians “with information about prenatal radiation exposure as an aid in counseling pregnant women”, states that “in all stages post-conception, radiation-induced non-cancer health effects are not detectable for fetal doses below about 0.10 Gy.” [5]

However, a significant increase in perinatal mortality was detected in Germany in 1987, one year after the Chernobyl accident [6]. Moreover, a significant association between perinatal mortality and the lagged cesium-137 burden of pregnant women was found in [6]. The effect increased disproportionately with the cesium burden. The increase in perinatal mortality in 1987 was later confirmed by Scherb et al. [7] who also reported a positive association of stillbirth rates with cesium soil deposition on a district level in Bavaria. According to UNSCEAR 1988 Annex D [8], Table 18, the effective dose from Chernobyl in the first year was 0.13 mSv in West Germany and 0.21 mSv in East Germany, i.e. more than two orders of magnitude smaller than the assumed threshold dose.

In Belarus, congenital abnormalities (CA) were significantly increased after Chernobyl in highly contaminated regions of Belarus [9]. The prevalence of all CA was greater in regions with a cesium soil contamination > 555 kBq/m^2^ than in a control region with soil contamination < 37 kBq/m^2^; the odds ratio was OR = 1.27 (95% CI: 1.00–1.61), *p* = 0.052. The effect was most pronounced for multiple malformations: OR = 1.75 (1.13–2.73), *p* = 0.013. There was no increase relative to the control region in regions with a cesium soil contamination between 37 and 555 kBq/m^2^ (OR = 1.03, *p* = 0.85). (Odds ratios determined by the present author from the data in Table 1 in [9] using multivariate logistic regression.)

**Table 1:** Effect of dispersion of radiation doses on response rates (parameters ${\sigma }_{1}$=0.3, ${\sigma }_{2}$=0.4)

Parameter ${\mu }_{1}$	0	0.2	0.3	0.4	0.6	0.8	1
Mean dose (mSv)	1.05	1.28	1.41	1.56	1.91	2.33	2.84
Adverse outcomes	1	12	31	73	291	1319	4552
Predicted^a^ $({\sigma }_{1}$=0.3)	1.8E-06	1.2E-05	2.9E-05	6.6E-05	3.2E-04	1.3E-03	4.6E-03
Predicted^a^ (${\sigma }_{1}$=0)	8.3E-09	1.3E-07	5.0E-07	1.7E-06	1.7E-05	1.3E-04	8.3E-04
Ratio^b^	219.0	88.5	57.9	38.9	18.6	9.7	5.5

^a^Predicted: Fitted values of response rates for ${\sigma }_{1}$=0.4 and ${\sigma }_{1}$=0, ^b^Ratio: Ratio of predicted values with (${\sigma }_{1}$=0.4) and without (${\sigma }_{1}$=0) dose dispersion

After the Fukushima accident in 2011, perinatal mortality in prefectures near the Fukushima Daiichi nuclear power station was significantly increased [10, 11]. The increase was characterized by periodic peaks in the spring seasons 2012–2017 [12].

A study of the prevalence of birth defects (BDs) in Germany that included questions on maternal occupational exposure to ionizing radiation within the first trimester of pregnancy found 4 infants with BDs in newborns of 29 exposed mothers (13.8%) compared to 161 infants in 3,787 births from unexposed mothers (4.3%), corresponding to RR = 3.2 (1.2–8.7) [13]. In a later study by these authors on congenital anomalies (CA) in the offspring of health workers potentially exposed to radiation in pregnancy, eight of 27 infants were diagnosed with CA (30%) compared with 6.2% of the comparison group [14].

The concept of a threshold dose is derived from results of animal experiments, mostly on mice, exposed during the period of organogenesis to rather high x-ray doses in the range of some Gray (Gy). Typical survival curves are of a shoulder type, i.e. with no discernable effect at low doses and a sharp decrease at doses of 1 Gy or higher (see Supplementary Material Fig. S1). According to ICRP 90 (ICRP 90), the maximum sensitivity in mice embryos is observed on days 7–8 post-conception (p.c.), the period of major organogenesis, which corresponds to 6–7 weeks p.c. in human embryos.

The above results from epidemiologic studies in human populations are incompatible with the existence of a threshold dose of 100 mSv. Here, a hypothesis is proposed presenting a dose–response relationship without threshold.

### Presentation of the hypothesis

#### H   ypothesis

The dose–response relationship for teratogenic radiation effects is a cumulative lognormal distribution without threshold if it is assumed that both radiation doses and radiosensitivities are random variables represented by lognormal probability density distributions.

The justification for using a lognormal distribution for radiosensitivity is based on the shape of the survival curves of irradiated experimental mice. The dose–response relationship can well be approximated by a cumulative lognormal distribution (see Supplementary Material, Fig. S2). Lognormal distributions are also well suited for modeling population doses after the Chernobyl accident (see Supplementary Material, Fig. S3).

The lognormal probability density function has the following analytical form:

$$f(x, \mu ,\sigma ) = 1/(x\sigma \sqrt{2\pi })\cdot exp(-{(\mathrm{log}(x)-\mu )}^{2}/2{\sigma }^{2}$$).

Here, variable $x$ is the dose (or dose rate) and µ and $\sigma$ are the two parameters defining the distribution. The parameter µ is the natural logarithm (log) of the median value of the distribution and $\sigma$ is the standard deviation. In the following, $f({x}_{1}, {\mu }_{1},{\sigma }_{1})$ denotes the distribution of doses and $g({x}_{2}, {\mu }_{2},{\sigma }_{2})$ the distribution of radiosensitivities.

For the following calculation, the term “radiosensitivity” is replaced by a dose-dependent individual repair capacity on the DNA level. An All-or-None approach is used, i.e. all radiation damage to the DNA (e.g. double-strand breaks) is repaired at doses (or dose rates) up to a critical dose (threshold dose) and fails above the threshold dose (dose rate). Then the distribution $g({x}_{2}, {\mu }_{2},{\sigma }_{2})$ refers to the distribution of individual threshold doses (dose rates) in an exposed population. For simplicity, dose will be used hereafter for either dose or dose rate.

A large number N of random radiation doses (${x}_{1}$) and threshold doses (${x}_{2}$) is generated by Monte Carlo simulation, based on the lognormal distributions $f({x}_{1}, {\mu }_{1},{\sigma }_{1})$ and $g({x}_{2}, {\mu }_{2},{\sigma }_{2})$, respectively. Each value of dose ${x}_{1}$ is compared with the respective value of the threshold dose ${x}_{2}$; in case ${x}_{1}$ is greater than ${x}_{2}$, the value of a counter (*n*) is increased by 1. After N comparisons, the final value of n, divided by N, is the proportion $\overline{y }$ = n/N of adverse outcomes. The related mean dose is $\overline{x }=\Sigma ({x}_{1})/N$. An adverse outcome can be, among other, spontaneous abortion, birth defect (congenital malformation), stillbirth, or perinatal mortality. In the following, the term “response” is used to denote an adverse outcome.

**Fig. 1:**
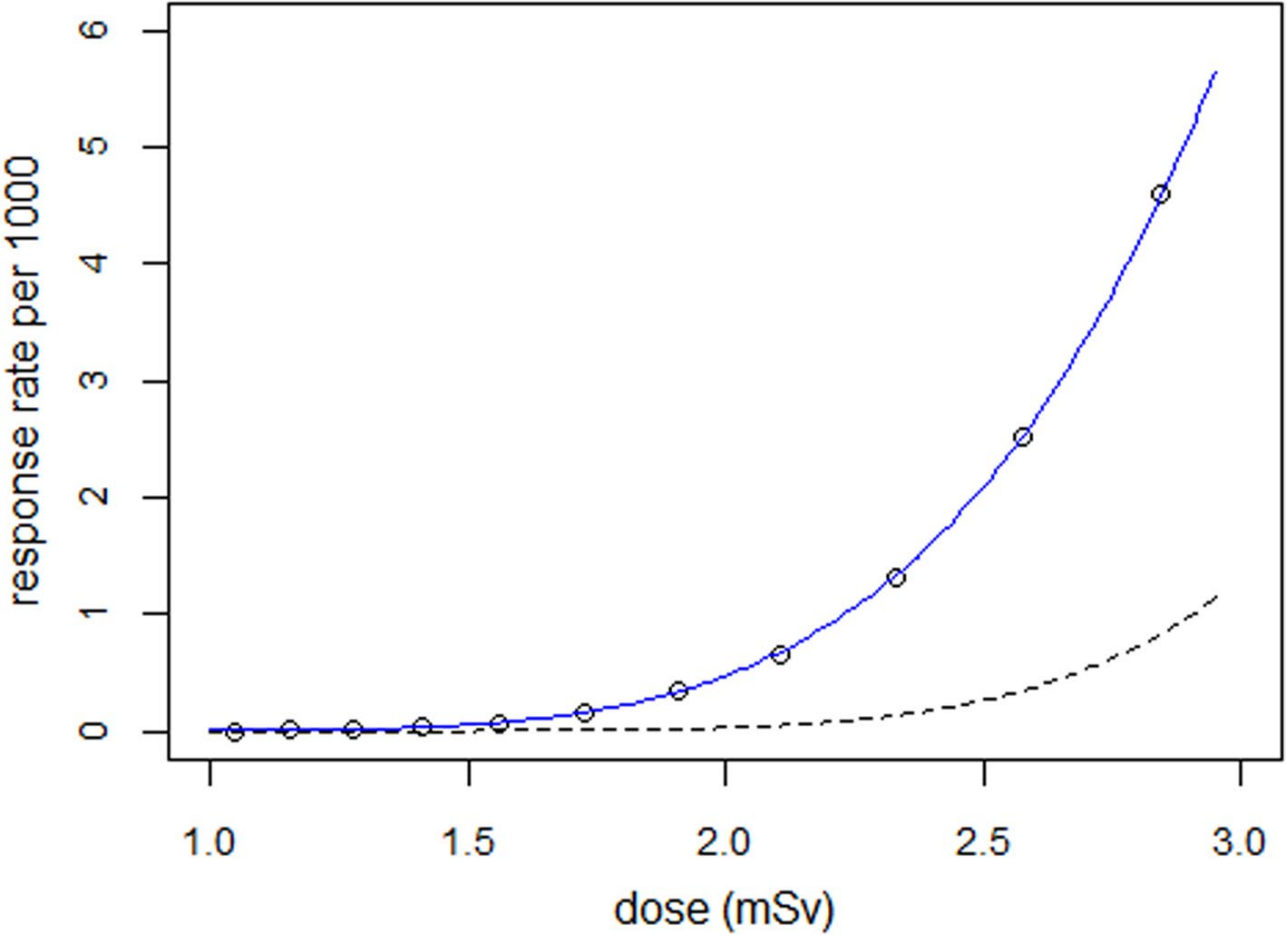
Simulated response rates as a function of mean radiation dose and result of regression with a cumulative lognormal distribution (blue line). The interrupted line shows the calculated dose–response curve for discrete doses (${\sigma }_{1}$= 0).

The above procedure is carried out for k = 11 values of parameter ${\mu }_{1}$ of the dose distribution with ${\mu }_{1}$ = 0, 0.1, …, 1.0. To display the shape of the dose–response curve, response rates $\overline{y }$[k] are plotted as a function of mean dose $\overline{x }$[k] (see Fig. 1). The dose–response relationship is determined by iteratively re-weighted non-linear regression of $\overline{y }$[k] on $\overline{x }$[k] with program nls() of statistical package R [15]. R was also used for Monte Carlo simulations and plotting. F-tests / t-tests are applied for statistical tests, and a *p*-value < 0.05 was considered statistically significant.

For the numerical calculation, the following values of the parameters were chosen: ${\sigma }_{1}$ = 0.3, ${\mu }_{2}$ = log(10) = 2.303, ${\sigma }_{2}$ = 0.4, and N = 1 million. To each of the 11 median doses $exp({\mu }_{1})$, mean doses $\overline{x }$[k] and mortalities $\overline{y }$[k] were calculated as described above. A cumulative lognormal function was used for the regression. The parameter estimates were µ = 2.331 ± 0.012 and σ = 0.494 ± 0.004. The result for µ agreed with ${\mu }_{2}$+${\sigma }_{1}^{2}$/2 = 2.348 within the limits of error. Similarly, the result for σ agreed with σ = $\sqrt{{\sigma }_{1}^{2}+{\sigma }_{2}^{2}}$ = $\sqrt{{0.3}^{2}+{0.4}^{2}}$  = 0.5. Thus, the mathematical form of the dose–response curve is likely to be a lognormal distribution with µ = ${\upmu }_{2}$+${\sigma }_{1}^{2}$/2 and σ = $\sqrt{{\sigma }_{1}^{2}+{\sigma }_{2}^{2}}$.

For comparison, a regression of response rates $\overline{y }$[k] on doses $\overline{x }$[k] was conducted with a power-law function of the form $\overline{y }$[k] = a·$\overline{x }$^b. The resulting power b was estimated as 6.79 ± 0.14. The model with the lognormal distribution, however, fitted the data better (deviance = 6.0, df = 9) than the power-law model (deviance = 37.7, df = 9).

The effect of non-linearity of the dose–response curve is very large: The response is 0.029 per 1000 at mean dose 1.41 mSv (${\mu }_{1}$= 0.3) and 4.6 per 1000 at mean dose 2.84 mSv (${\mu }_{1}$= 1.0), an increase by a factor of 160 for a doubling in dose (see Table 1, fourth row). The effect of the width of the dose distribution (parameter ${\sigma }_{1})$ on response rates can also be very large. The predicted response rate at a mean dose of 2.84 mSv is 0.83 per 1000 for ${\sigma }_{1}$= 0 (no dispersion) and 4.6 per 1000 for ${\sigma }_{1}$= 0.3, a ratio of 5.5 (see Table 1, last column). The effect is greater for smaller doses; at 1.41 mSv (${\mu }_{1}$= 0.3) the ratio is 58.

To check whether the formula for the standard deviation, i.e. $\sigma$ = $\sqrt{{\sigma }_{1}^{2}+{\sigma }_{2}^{2}}$, can be verified, 10 consecutive simulations were performed. The expected value for $\sigma$ is $\sqrt{{0.3}^{2}+{0.4}^{2}}$ = 0.5. Table 2 shows the estimates of parameter $\sigma$ resulting from regressions of the response rates, together with the standard errors of estimate (SE). The third row shows the z-scores, i.e. the deviations of the estimates of $\sigma$ from the expected value (0.5) divided by SE: z = ($\sigma$-0.5)/SE. The average deviation was -0.0017 ± 0.0016, thus confirming the validity of $\sigma$ = $\sqrt{{\sigma }_{1}^{2}+{\sigma }_{2}^{2}}$.

**Table 2:** Estimated standard deviations $\sigma$ with standard errors (SE) and z-scores

Estimate	0.494	0.502	0.501	0.504	0.502	0.508	0.497	0.486	0.492	0.497
SE^a^	0.005	0.005	0.003	0.004	0.006	0.005	0.006	0.007	0.005	0.007
z-score^b^	-1.04	0.38	0.23	1.18	0.37	1.42	-0.48	-2.17	-1.70	-0.44

^a ^*SE* standard error of estimate

^b ^z-score: z = ($$\sigma$$-0.5)/SE

To investigate the effect of the width of the dose distribution on the response rates, the dose–response relationship was determined with standard deviation ${\sigma }_{1}$= 0.4 instead of ${\sigma }_{1}$= 0.3. The parameters for the distribution of radiosensitivities (threshold doses) were retained (${\mu }_{2}$= log(10) and ${\sigma }_{2}$= 0.4).

**Fig. 2:**
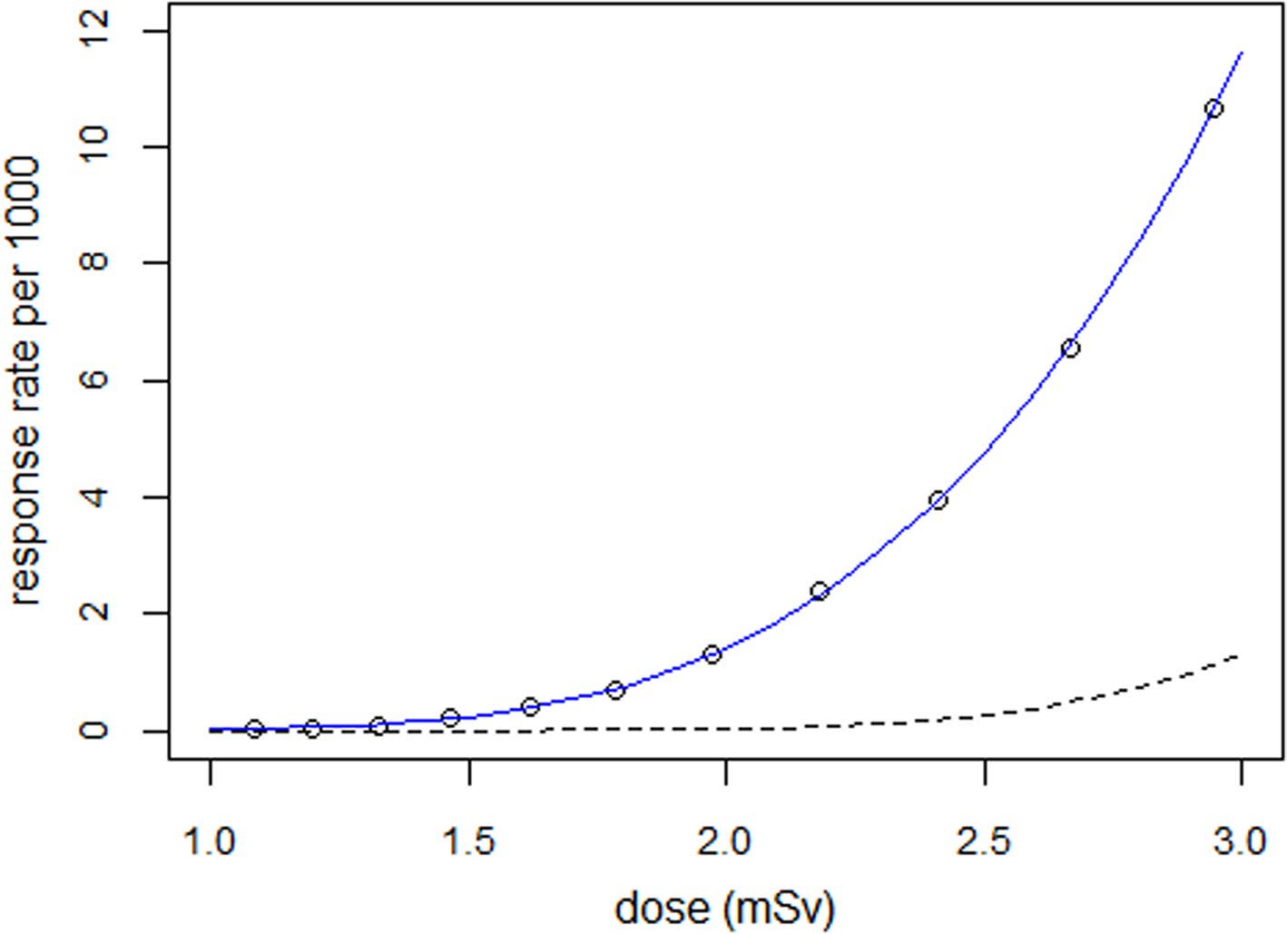
Simulated response rates as a function of mean radiation dose for a dose distribution with parameter ${\sigma }_{1}$= 0.4, and regression line. The interrupted line shows the calculated dose–response curve for discrete doses (${\sigma }_{1}$= 0)

Figure 2 shows the simulated response rates with ${\sigma }_{1}$= 0.4 and the regression line. For comparison, the predicted dose–response curve for ${\sigma }_{1}$= 0 is added. A regression of the data with power-law yields a power of 5.42 (5.17–5.70). Again, the model with the lognormal distribution fitted the data better (deviance = 4.6, df = 9) than the power-law model (deviance = 106.7, df = 9).

The response rate was 10.6 per 1000 at 2.95 mSv (see Table 3, ${\mu }_{1}$= 1.0) for ${\sigma }_{1}$= 0.4 which compares to 4.6 per 1000 at 2.84 mSv (see Table 1) for ${\sigma }_{1}$= 0.3. The difference is even greater at lower dose with 0.2 per 1000 at 1.46 mSv (Table 3, ${\mu }_{1}$= 0.3) compared to 0.03 per 1000 at 1.41 mSv (see Table 1). The last 3 rows of Table 3 contain the predicted response rates for ${\sigma }_{1}$= 0.4 (fourth row) and ${\sigma }_{1}$= 0 (discrete doses, fifth row), and the ratios of the numbers in the fourth and fifth row (last row); the ratio is 9.5 at a mean dose of 2.95 mSv (${\mu }_{1}$= 1.0) and 269 at 1.46 mSv (${\mu }_{1}$= 0.3).

**Table 3:** Effect of dispersion of radiation doses on response rate (parameters ${\sigma }_{1}$=0.4, ${\sigma }_{2}$=0.4)

Parameter ${\mu }_{1}$	0	0.2	0.3	0.4	0.6	0.8	1.0
Mean dose (mSv)	1.08	1.32	1.46	1.62	1.97	2.41	2.95
Adverse outcomes	21	103	228	393	1299	4034	10,633
Predicted^a^ $({\sigma }_{1}$=0.4)	2.5E-05	1.0E-04	2.1E-04	4.0E-04	1.3E-03	4.0E-03	1.1E-02
Predicted^a^ (${\sigma }_{1}$=0)	1.4E-08	2.1E-07	7.7E-07	2.6E-06	2.5E-05	1.9E-04	1.1E-03
Ratio^b^	1803	492	269	152	53.5	21.2	9.5

^a^ Predicted: Fitted values of response rates for ${\sigma }_{1}$=0.4 and ${\sigma }_{1}$=0

^b^ Ratio: Ratio of predicted values with (${\sigma }_{1}$=0.4) and without (${\sigma }_{1}$=0) dose dispersion

### Testing of the hypothesis

**Fig. 3:**
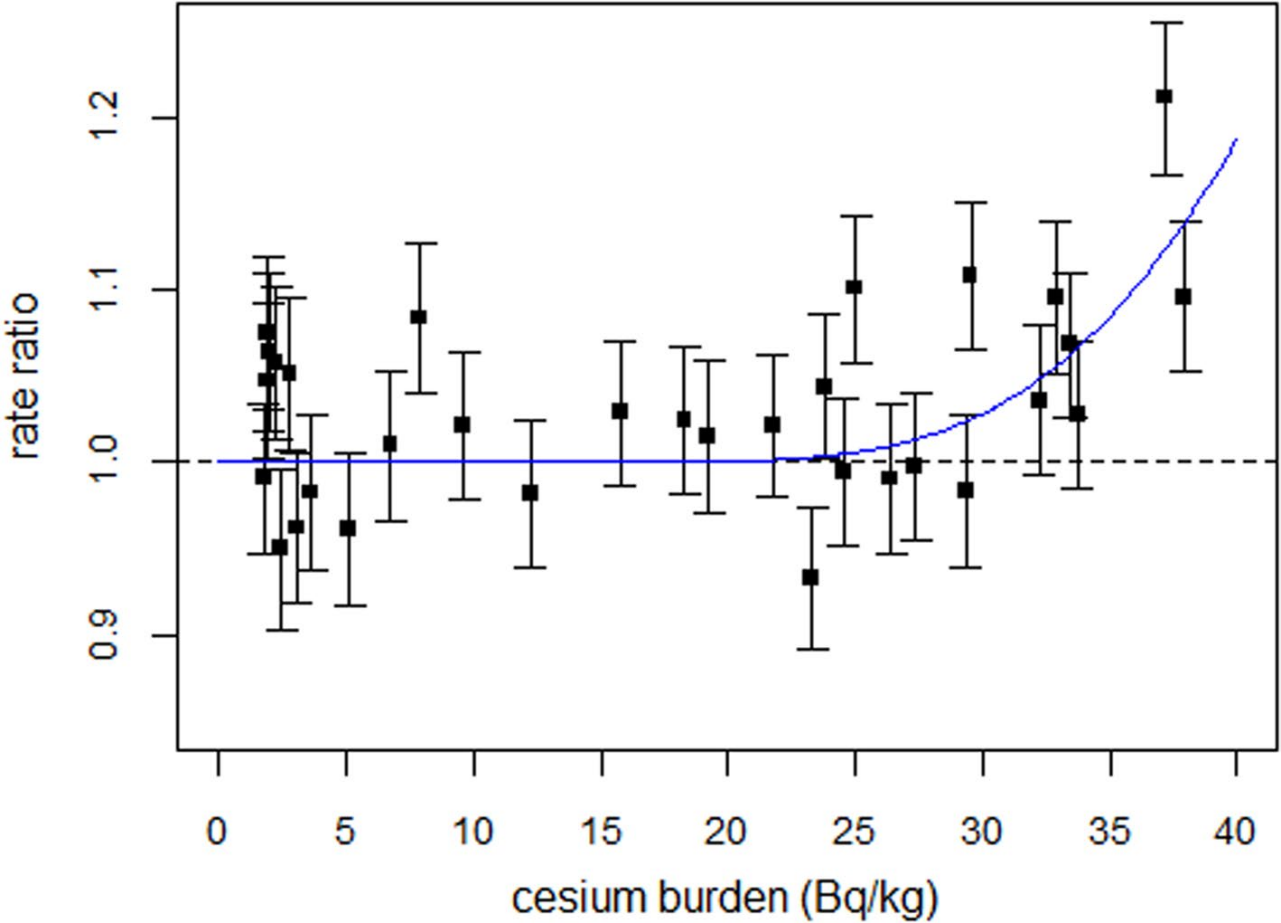
Ratios of observed perinatal mortality rates to those predicted with the reduced model as a function of cesium burden of pregnant women, and result of regression with a cumulative lognormal distribution. The error bars are one standard deviation

To test whether the dose–response relationship derived above can be applied to fit real data, German perinatal mortality data after Chernobyl are used [6]. The effect of cesium burden (Bq/kg) in pregnant women on perinatal mortality seven months later is modeled by a cumulative lognormal distribution. The estimates of the parameters were µ = 3.97 ± 0.19 and σ = 0.30 ± 0.14. The effect of the cesium term was highly significant (*p* < 0.001, F-test). Regression with power-law yielded power 6.4 (2.0–16.5). Figure 3 shows the ratios (rate ratios) of observed perinatal mortality rates and the rates predicted with the reduced model, i.e., the predicted rates without the cesium term. A “practical” threshold is found for a cesium burden of about 20 Bq/kg.

### Implications of the hypothesis

In the present study, the shape of the dose–response relationship for teratogenic effects is derived from two assumptions: Both radiosensitivities and radiation doses for an exposed population are random variables. Using lognormal density functions for both dose distribution and radiosensitivities, a cumulative lognormal distribution with no threshold is obtained as the dose–response relationship.

Although the main objective of the present study was to demonstrate the absence of a threshold dose for teratogenic radiation effects, the question arises as to why adverse health effects in mice are detected only at radiation doses on the order of Gray, whereas such effects are observed in human populations at doses 1000-fold lower. In Munich (Germany), the calculated Cs-137 exposure of pregnant women in the first year after the Chernobyl accident was less than 40 Bq per kg body weight (see Fig. 3). 40 Bq per kg corresponds to a dose rate of 0.03 µGy/h, or about 0.05 µGy/h if the dose rate of Cs-134 is added. This is only half the dose rate of natural gamma background radiation (0.08 µGy/h).

As shown above, radiation dose dispersion can have a large impact on pregnancy outcomes, especially at very low doses. Discrete doses are administered in mouse experiments, whereas individual doses may vary widely in human populations exposed to radiation from nuclear accidents. In addition, the strains of mice used for experimental purposes may have low genetic variability. Therefore, the effect of dispersion may be negligible in mouse experiments.

Acceptance of the hypothesis means that studies of adverse health effects following in-utero exposure to low doses of ionizing radiation should not be discarded primarily because they contradict the concept of a threshold dose for teratogenic effects.

## Supplementary Information

**Additional file 1. **Supplementary material

## Data Availability

Not applicable
