# Supplementary material for: A hypothesis to derive the shape of the dose–response curve for teratogenic radiation effects
Source: Environ Health. 2022 Feb 10;21:25. doi: 10.1186/s12940-022-00837-z (PMC8829992; doi:10.1186/s12940-022-00837-z)
Supplement: Supplementary file 1 — Additional file 1. Supplementary material [file 12940_2022_837_MOESM1_ESM.docx]

Supplementary material

**1. Dose-response relationship in irradiated experimental mice**

The concept of a threshold dose is derived from results of animal experiments, mostly on mice, exposed during the period of organogenesis to rather high x-ray doses in the range of some Gray (Gy). Typical survival curves are of a shoulder type, i.e. with no discernable effect at low doses and a sharp decrease at a dose of 1 Gy or higher (see Figure S1).


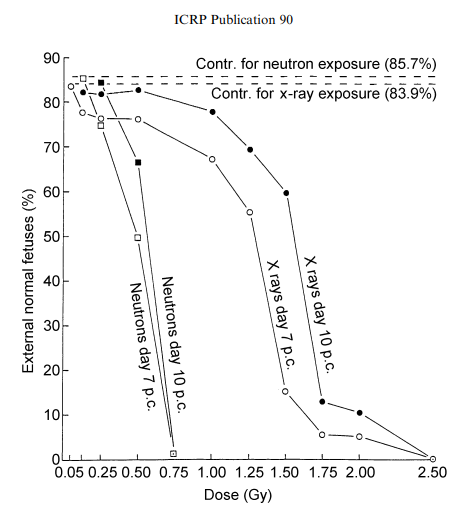


Figure S1. Live fetuses without external defects (%) in mice on day18 post-conception (p.c.) after acute exposure to x rays or neutrons on days 7 or 10 p.c. Percentages are related to the total implantation number of each group (Konermann 1987 with permission from Elsevier).
From: ICRP 90, Fig. 3.3.

To check whether the dose-response relationship can be modeled by a lognormal distribution, the data points in Figure 1 (which show the proportion of mice without birth defects after irradiation with x-rays on day 7 p.c.) are transformed to show the proportion of mice with birth defects (see Figure 2). The solid line in Figure 2 is the result of regression of the proportion of birth defects with a cumulative lognormal distribution; the estimates of the parameters were µ = 0.29 ± 0.02 (which corresponds to a median dose of 1.34 Gy) and σ = 0.17 ± 0.03. The dotted line in Figure 2 shows the level of birth defects in a non-exposed control group. Thus, the dose-response relationship can well be approximated by a cumulative lognormal distribution.


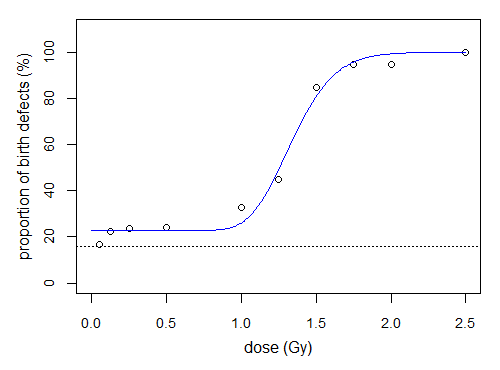

Figure S2. Proportion of birth defects in mice at term after x-ray exposure on day 7 p.c.
(Data taken from Figure 1, x-rays, day 7 p.c.)

**2. Dose distribution in a human population exposed to Chernobyl fallout**

To investigate whether a lognormal density function can also be applied to the distribution of doses in a population exposed to Chernobyl fallout, measurements of the cesium burden in pregnant women from the Ukrainian region Rivne (Rovno in Russian) in 2011-2013 were used (Wertelecki 2016).

Figure 3 shows the frequency of cesium whole-body counts per kg body weight and the result of regression with a lognormal density distribution. The median of the cesium burden was estimated as 29.9 Becquerel (Bq) per kg body weight, and the estimate of the standard deviation was 0.63. Thus, the dose distribution can well be approximated by a lognormal density function.


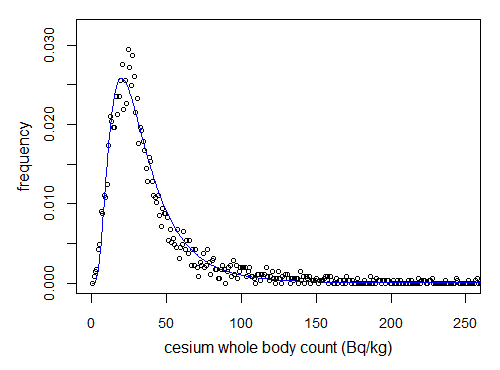

Figure S3. Distribution of cesium whole-body counts in pregnant women from Rivne (Ukraine) and result of regression with a lognormal density function.

Reference

Wertelecki W, Koerblein A, Ievtushok B, Zymak-Zakutnia N, Komov O, Kuznietsov I, Lapchenko S, Sosyniuk Z. Elevated congenital anomaly rates and incorporated cesium-137 in the Polissia region of Ukraine. Birth Defects Res A Clin Mol Teratol. 2016 Mar;106(3):194-200. doi: 10.1002/bdra.23476. Epub 2016 Feb 12. PMID: 26871487.

**3. R-script for Monte Carlo simulation and regression**

# Monte Carlo simulation

N=1E6

mu1 <- (0:10)/10

sigma1 <- 0.3

mu2 = log(10)

sigma2 <- 0.4

O <- c(rep(0,11))

dose <- c(rep(0,11))

for(k in 1:11)

{

x1 <- rlnorm(N,meanlog=mu1[k],sdlog=sigma1) # random doses

x2 <- rlnorm(N,meanlog=mu2,sdlog=sigma2) # random sensitivities

n=0

for(i in 1:N)

{

if(x1[i]>x2[i]) n <- n+1

}

print(n)

O[k] <- n

dose[k] <- mean(x1)

}

rate <- O/N

# regression with lognormal distribution

var <- rate*(1-rate)/N

fm <- nls(rate1~plnorm(dose,meanlog=c1,sdlog=c2),weights=1/var,

start=list(c1=2.3,c2=0.5))

# reweighting

Q=0

while(abs(Q-deviance(fm)) > 0.001)

{

Q = deviance(fm)

fit <- fitted(fm)

var <- fit*(1-fit)/N

fm <- nls(rate~plnorm(dose,meanlog=c1,sdlog=c2),weights=1/var,

start=list(c1=2.3,c2=0.5))

print(deviance(fm))

}

summary(fm)
